# Glucose-6-phosphate dehydrogenase deficiency in the Han Chinese population: molecular characterization and genotype–phenotype association throughout an activity distribution

**DOI:** 10.1038/s41598-020-74200-y

**Published:** 2020-10-13

**Authors:** Ying He, Yinhui Zhang, Xionghao Chen, Qiong Wang, Lifen Ling, Yuhong Xu

**Affiliations:** 1 Department of Laboratory Medicine, The Eighth Affiliated Hospital, Sun Yat-Sen University, Shenzhen, Guangdong Province, China; 2 Maternity and Children, Healthcare Hospital of Futian, Shenzhen, Guangdong Province, China; 3 Department of Clinical Pharmacy, The Eighth Affiliated Hospital, Sun Yat-Sen University, Shenzhen, Guangdong Province, China

**Keywords:** Medical research, Molecular medicine

## Abstract

Glucose-6-phosphate dehydrogenase (G6PD) deficiency is a common hereditary disorder in China. The existing prevalence and molecular epidemiology of G6PD deficiency in China were geographically limited. In this study, the spectrum of G6PD gene mutations was well characterized in a large and diverse population all over the country; and the correlation of genotype and enzyme activity phenotype was explored for the first time. The results showed that the overall prevalence of G6PD deficiency in China was 2.10% at the national level. The top six common mutations were c.1388 G>A, c.1376 G>T, c.95 A>G, c.392 G>T, c.871 G>A and c.1024 C>T, accounting for more than 90% of G6PD deficient alleles. Compound mutation patterns were frequently observed in females with severe deficiency. The distribution of G6PD activities depended on the type of mutation patterns and genders. Hemizygote, homozygote, and compound heterozygote were predominantly associated with severe G6PD deficiency, whereas heterozygotes with single mutation mainly presented moderate enzyme deficiency. A significant gap between G6PD activities in hemizygous and normal males was observed, and yet, the overall distribution of that in females carrying missense mutations was a continuum from G6PD severely deficient to normal. This is the first report of discussing the association between G6PD genetic variants in the Chinese and enzyme activity phenotypes.

## Introduction

G6PD deficiency in human red blood cells is the most common human enzyme defect with an estimated 400 million population affected worldwide. The prevalence of G6PD deficiency is highly variable around the world, even in different parts of the same country. The highest prevalence is reported in Africa, southern Europe, the Middle East, Southeast Asia, and Mediterranean countries. Migration and resettlement have an impact on the distribution of this disorder^1–3^. G6PD is encoded by the G6PD gene located in the telemetric region of the long arm of the X-chromosome (Xq28). Therefore the inheritance of G6PD deficiency shows a typical X-linked pattern. Males with this disorder will always be hemizygous, while females may be heterozygous or more rarely homozygous^1^. Heterozygous females are genetic mosaics as a result of X-chromosome inactivation and may have normal or deficient enzyme activities, depending on the relative proportion of G6PD deficient red cells. Mutations distributed throughout the G6PD gene can result in amino acid substitutions, and subsequently protein variants with different levels of enzyme activity^2,3^. More than 200 mutations in the G6PD gene have been described, most of which are single-base substitutions^4^.

G6PD variants have been classified into five classes by the World Health Organization (WHO) based on the residual enzyme activity and clinical manifestations^3^. In general, individuals with G6PD deficiency will remain clinically asymptomatic throughout all or most of their lives. However, some individuals with this disorder may develop acute haemolysis triggered by the ingestion of fava beans, oxidative medications, infections, and other unknown exogenous agents. In newborns, G6PD deficiency is associated with an increased risk of neonatal jaundice and bilirubin encephalopathy in some cases. The most effective management for G6PD deficiency is to prevent haemolysis by avoiding exogenous oxidative stress, which requires people to become aware of their status of deficiency^2,3^.

G6PD deficiency is also a common genetic disorder in China. There are at least 35 different mutations that have been identified in the Chinese population^5^. Previous studies demonstrated that the prevalence of G6PD deficiency in China is highly variable among different regions or ethnicities^6,7^, and higher prevalence has been reported in southern China^7–10^, such as Guangdong, Guangxi, Yunnan, Hainan provinces, and so on. The newborn screening program is intensively performed in the south region of China and is expanding to other regions, with the highly variable cutoff value from 2–10 IU/g Hb in different laboratories^5,11^. Although the spectrum of mutations of G6PD deficiency in the Chinese population was surveyed in recent years^12–15^, these studies included a small number of people and were geographically limited. Information regarding the molecular characterization of G6PD in China was fragmentary hitherto. Few nationwide molecular epidemiological survey of G6PD deficiency was reported^5^. And so far, no studies on the correlation between specific G6PD mutations and G6PD activity phenotype are reported in the Chinese population. Therefore, there is a need to characterize the spectrum of G6PD gene mutations in the large and diverse population of the country and the distribution of G6PD activity for various variants in the Chinese population.

## Results

### Study population and prevalence of G6PD deficiency

During the three years’ prospective study of G6PD deficiency, 74,114 healthy adults were tested, 36,909 of whom were females and 37,205 were males. The age ranged from 19 to 45 years old, with a mean age of 28.97 ± 4.68 years. All subjects were Han ethnic Chinese and originated from 21 different provinces and municipalities of China, mainly from Guangdong, Hunan, Hubei, Jiangxi, Sichuan, Chongqing, and Guangxi, accounting for 74.6% of all cases. The enrolled subjects and prevalence of G6PD deficiency in subgroup populations were listed in Table 1. Among these subjects, a total of 1555 (578 females and 977 males) were found to be G6PD deficient with an overall prevalence of 2.1%, and that in females and males was 1.6% and 2.6%, respectively. The ratio of males and females was 1.6:1. There was a significant difference in the prevalence of G6PD deficiency between genders (*χ*^2^ = 104.12, p = 0.000).

**Table 1:** The prevalence of G6PD deficiency and the distribution of common G6PD mutations in subpopulations from 21 different native places.

Region	Native place	Enrolled subjects	Deficient Subjects	Incidence rate (%)	Subjects for mutation detection	Main mutation distribution							
						c.1376 G>T	c.1388 G>A	c.95 A>G	c.392 G>T	c.871 G>A	c.1024 C>T	Others	None
North China	Liaoning	891	0	0	0	–	–	–	–	–	–	–	–
	Shandong	1210	2	0.17	0	–	–	–	–	–	–	–	–
	Shanxi	1124	2	0.18	2	2	0	0	0	0	0	0	0
	Jilin	925	2	0.22	0	–	–	–	–	–	–	–	–
	Henan	2457	8	0.33	2	1	1	0	0	0	0	0	0
	Tianjin	1207	2	0.17	0	–	–	–	–	–	–	–	–
South China	Jiangsu	1408	3	0.21	1	0	1	0	0	0	0	0	0
	Anhui	1041	4	0.38	0	–	–	–	–	–	–	–	–
	Shanghai	978	10	1.02	1	0	1	0	0	0	0	0	0
	Fujian	1437	15	1.04	5	1	2	1	0	1	0	0	0
	Guangdong	18,252	678	3.71	266	84	94	32	19	19	13	13	11
	Guangxi	3421	117	3.42	35	9	14	6	4	2	1	1	0
	Hubei	8247	102	1.24	47	12	14	10	3	3	4	3	0
	Hunan	9482	107	1.13	45	12	18	8	3	3	3	1	2
	Jiangxi	4781	109	2.28	35	8	12	3	3	1	2	3	3
	Sichuan	5917	103	1.74	41	12	15	6	3	1	1	2	1
	Chongqing	5189	133	2.56	46	15	15	7	2	2	3	1	3
	Guizhou	1228	35	2.85	11	3	2	2	1	1	1	1	0
	Hainan	2385	74	3.10	18	8	4	2	1	2	1	0	0
	Yunnan	1264	44	3.48	16	4	4	2	1	4	1	0	0
	Zhejiang	1270	5	0.39	0	0	0	0	0	0	0	0	0
Total		74,114	1555	2.10	571	171	197	79	40	39	30	25	20

A short dash line presented that the detection of G6PD mutations was not performed in this subpopulation due to no subjects selected randomly.

### Mutation spectrum of G6PD deficiency

During the second study year, 571 unrelated individuals (224 females and 347 males) were identified with G6PD deficiency and underwent mutation testing. The subjects for mutation detection and the distribution of the G6PD mutation in subpopulations from different native place was shown in Table 1. Among these G6PD deficient individuals, 547 (215 females and 332 males) had at least one G6PD gene mutation by PCR-RDB assay. The remaining individuals (9 females and 15 males) had no identifiable mutation, four males of which were found to have mutations by further DNA sequence analysis. As a result, a total of eleven missense mutations and one polymorphism (c.1311C>T) were identified from 551 individuals (215 females and 336 males) with G6PD deficiency in this study. All of the mutations detected in our study were previously reported in the Chinese population. Among 215 G6PD deficient females with mutations, we found 22 (10.2%) homozygotes and 193 heterozygotes, including 30 compound heterozygotes (containing two missense mutations). Homozygotes and compound heterozygotes mainly involved the mutation of c.1376C>T or c.1388G>A.

The mutations of c.1388G>A and c.1376G>T were two dominant variants in the Han Chinese population with a frequency of more than 64% in total, followed by the mutations of c.95A>G, c.392G>T, c.871G>A and c.1024C>T, with a minimum frequency of 5%, respectively. 65 (11.4%) G6PD deficient individuals were identified with the polymorphic mutation of c.1311C>T, 11 (6 females and 5 males) of whom with only c.1311C>T polymorphism, but no pathogenic mutations. In all 39 subjects with c.871G>A mutation, the polymorphism of c.1311C>T was identified concomitantly. The number and frequency of various mutation types in subjects with G6PD deficiency were presented in Table 2.

**Table 2:** Distribution of different G6PD mutation patterns and the corresponding enzyme activities observed from 571 subjects with G6PD deficiency.

Mutation pattern	Hemi	Enzyme activity	Hetero	Enzyme activity	Homo	Enzyme activity	Total*	Frequency (%)
c.95 A>G	49	0.81 ± 0.63, 0.02–2.73	18	6.03 ± 2.15, 0.60–9.17	1	0.04	68(2)	11.91
c.392 G>T	20	1.85 ± 0.78, 0.76–3.16	13	5.84 ± 2.08, 1.60–9.32	1	1.01	34	5.95
c.493 A>G	1	0.64	0	–	0	–	1	0.18
c.517 T>C^#^	1	1.39	0	–	0	–	1	0.18
c.592 C>T	0	0	3	6.23 ± 1.58, 4.45–7.47	0	–	3	0.52
c.871 G>A	21	0.56 ± 0.53, 0.06–2.17	12	5.78 ± 2.72, 0.51–9.72	1	5.54	34(34)	5.95
c.1004 C>A	1	1.58	0	–	0	–	1(1)	0.18
c.1024 C>T	14	2.58 ± 0.83,1.52–4.16	13	5.81 ± 1.80, 1.14–7.72	0	–	27(2)	4.73
c.1311 C>T	5	2.55 ± 3.22, 0.06–8.30	6	4.63 ± 2.77, 0.24–7.68	0	–	11	1.93
c.1360 C>T^#^	3	0.68 ± 0.39, 0.24–0.96	4	4.93 ± 2.80, 2.13–8.76	0	–	7(2)	1.22
c.1376 G>T	95	0.47 ± 0.38, 0.01–2.03	52	6.11 ± 2.48, 0.42–9.98	8	0.56 ± 0.53, 0.07–1.38	155(3)	27.14
c.1388 G>A^#^	126	0.66 ± 0.49, 0.04–2.82	42	6.58 ± 2.10, 0.00–9.97	11	1.52 ± 1.01, 0.53–3.41	179(5)	31.35
c.95 A>G/ c.1376 G>T	0	–	6	0.47 ± 0.28, 0.11–0.79	0	–	6	1.05
c.95 A>G/ c.1388 G>A	0	–	4	1.13 ± 0.23, 0.90–1.40	0	–	4	0.70
c.95 A>G/ c.871 G>A/ c.1311 C > T	0	–	1	1.01	0	–	1	0.18
c.392 G>T/c.1376 G>T	0	–	1	1.13	0	–	1	0.18
c.392 G>T/c.1388 G>A	0	–	5	2.20 ± 1.56, 0.73–4.62	0	–	5	0.88
c.871 G>A / c.1376 G>T/ c.1311 C > T	0	–	2	0.36 ± 0.06, 0.32–0.40	0	–	2	0.35
c.871 G>A / c.1388 G>A/ c.1311 C > T	0	–	2	1.62 ± 0.63, 1.18–2.07	0	–	2	0.35
c.1024 C>T/c.1376 G>T	0	–	2	1.68 ± 1.63, 0.52–2.83	0	–	2	0.35
c.1024 C>T/c.1388 G>A	0	–	1	2.39	0	–	1	0.18
c.1360 C>T/c.1388 G>A	0	–	1	0.08	0	–	1	0.18
c.1376 G>T/c.1388 G>A	0	–	5	0.65 ± 0.52, 0.14–1.36	0	–	5	0.88
Mutation unknown	11	2.91 ± 2.77, 0.12–8.30	9	6.03 ± 2.15, 0.60–9.17	0	–	20	3.50
Total	347		202		22		571	100.0

The abbreviation Hemi, Hetero and Homo represented male hemizygote, female heterozygote and female homozygote, respectively. The enzyme activity was shown as mean ± SD and range. # Results of four male hemizyotes were from sequencing analysis, including c.517 T>C (1), c.1360 C>T (1) and c.1388 G>A (2). * In this column, the number in the bracket represented cases carrying c.1311 C>T polymorphism.

A total of 952 subjects (477 females and 475 males) with normal G6PD activity (> 10 IU/g Hb) were randomly selected for mutation analysis by PCR-RDB assay. The polymorphism of c.1311C>T was found in 78 females and 11 males, respectively. The pathogenic mutation was identified in 11 females, involving c.1376C>T (4), c.1388G>A (3), c.95A>G (2), c.392G>T (1) and c.1024C>T (1), while no pathogenic mutation was found in males.

### Correlations between genotype and G6PD activity phenotype

For all subjects with G6PD deficiency, the means and standard deviations, as well as the ranges of enzyme activities for various types of deficiency variants and non-variants, were presented in Table 2. In the hemizygous group, enzyme activity was very low, ranging from 0.01 to 4.16 IU/g Hb, whereas in the heterozygous group ranged between 0.01 and 9.98 IU/g Hb. The median value of G6PD activity in deficient females was approximately tenfold more than that in deficient males (4.89 ± 2.94 vs 0.79 ± 0.74 IU/g Hb, p = 0.000). The mean value of G6PD activity in normal females and males was 18.01 ± 3.37 (10.77–34.94) and 15.49 ± 2.67 (10.01–30.20) IU/g Hb, respectively. Twenty G6PD deficient subjects tested negative for the screened mutations had a mean activity of 2.91 ± 2.77 IU/g Hb for males and 6.79 ± 2.61 IU/g Hb for females, respectively. This group was not included in genotype and phenotype analysis since their mutation status could not be defined clearly.

**Figure 1:**
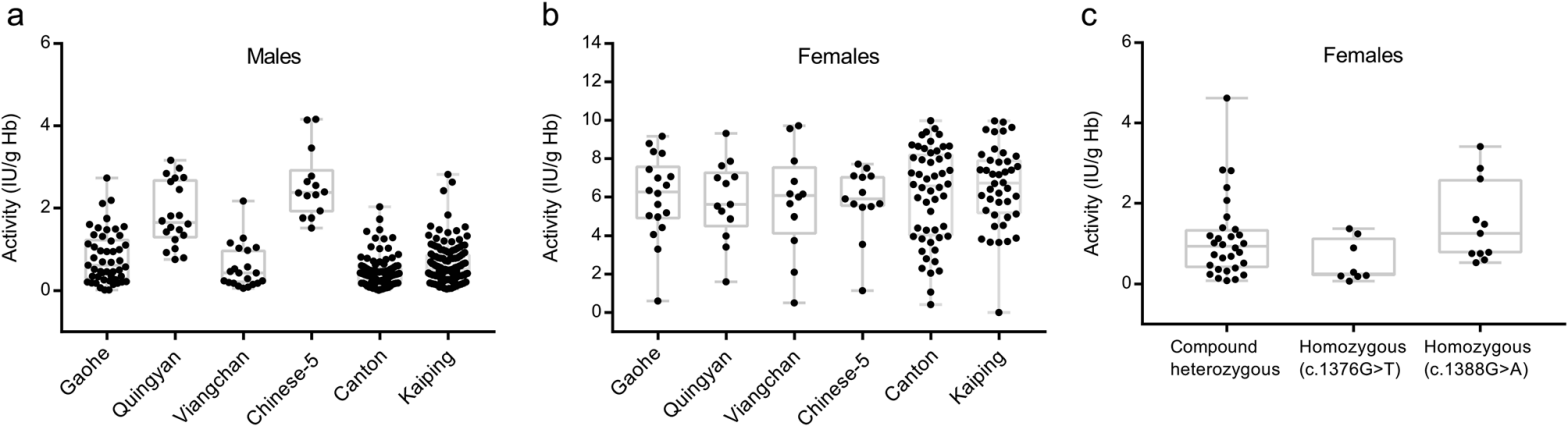
Distribution of G6PD activities by mutation types. Only mutation types, in which there were more than eight representatives, were shown. (**a**) G6PD activities of hemizygous males with various variants. (**b**) G6PD activities of heterozygous females with a single of different variant. (**c**) G6PD activities of compound heterozygous females, and homozygous females with c.1376G>T (Canton) and c.1388G>A (Kaiping), respectively.

For every variant, the distribution range of G6PD activity in hemizygous males was much narrower than that in heterozygous females, and the mean value of enzyme activity in hemizygous males was significantly lower than that in heterozygous females (p = 0.000) (Table 2). As shown in Fig. 1a,b, the enzyme activities in hemizygous mutants were significantly different between most of these variants (p values < 0.01), and the variant of Canton (c.1376G>T) had the lowest G6PD activity mean value, followed by Viangchan (c.871G>A), Kaiping (c.1388G>A) and Gaohe (c.95A>G). The variation of G6PD activities between different variants was also observed in single heterozygous females (carrying single nucleotide substitutions), but no significant difference between these variants was found (p-values from 0.184 to 0.999). The level of enzyme activity in homozygous and compound heterozygous females ranged from 0.04–5.54 IU/g Hb and 0.08–4.62 IU/g Hb, respectively (Table 2). We did not find a significant difference in G6PD activity between compound heterozygous and homozygous females (p = 0.934), but a significant difference in enzyme activity between homozygous females with c.1388G>A and those with c.1376G>T was observed (p = 0.020), as illustrated in Fig. 1c. On the whole, despite a certain overlap at the low end of G6PD activities in females, G6PD activity in single heterozygous females was significantly higher than that in hemizygous males with the same mutation (p = 0.000). There was no significant difference in G6PD activity between homozygous females and hemizygous males (1.06 ± 0.93 vs 0.79 ± 0.74 IU/g Hb, p = 0.122), while the comparison of activity values between compound heterozygous females and hemizygous males showed some differences (p = 0.046).

**Figure 2:**
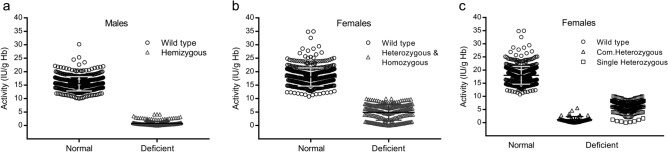
G6PD activity in all subjects divided into those without and those with G6PD deficiency. (**a**) G6PD activities in all-male subjects; (**b**) G6PD activities in all-female subjects. Eleven females carrying the G6PD gene mutation but with normal G6PD activities (12.63–27.15 IU/g Hb) were not illustrated in this deficient group. (**c**) G6PD activities in females without deficiency, with compound mutations, and with a single mutation, respectively.

In terms of G6PD activity, the male subjects could be divided into two separate groups: normal males and G6PD deficient males (Fig. 2a). The median value in deficient males was approximately 5.1% of that observed in normal males (p = 0.000). In heterozygous females, the median G6PD activity was about 30.1% of that seen in normal females (p = 0.000). There was no indiscernible boundary in enzyme activity between normal and deficient females, as illustrated in Fig. 2b. It was worth noting that, in compound heterozygous females, the median value of G6PD activity was only 5.1% of that observed in normal females (1.18 ± 1.14 vs 18.01 ± 3.37 IU/g Hb, p = 0.000). Compound heterozygous females could be also clearly distinguished from normal females by G6PD activity (Fig. 2c).

We classified the G6PD activities according to the WHO guideline. The median value (15.96 IU/g Hb) of G6PD activities in normal males considered as the normal G6PD activity. Distribution and the median value of G6PD activities according to the gender and genetic variants were shown in Table 3. For most variants in hemizygous males, the classification of enzyme activity collapsed into the expected class, which was consistent with the WHO classification. Nevertheless, for hemizygous males with the variant of Quingyan (c.392G>T), approximately 45% of the cases fell into class II, and 55% fell into class III, respectively. In single heterozygous females, the activities for every variant mainly fell into class III. Nevertheless, in homozygous and compound heterozygous mutants, roughly 80% of activities were under 1.6 IU/g Hb, being classified as class II.

**Table 3:** Classification and distribution of G6PD activities in 551 deficient subjects based on the WHO guideline. Note: Data were shown as number (percentage, mean value of enzyme activity).

Mutation pattern	WHO class	Actual classification for males with mutation			Actual classification for females with mutation		
		II (≤ 1.6 IU/g Hb)	III (1.6–9.6 IU/g Hb)	IV (9.6–24.0 IU/g Hb)	II (≤ 1.6 IU/g Hb)	III (1.6–9.6 IU/g Hb)	IV (9.6–24.0 IU/g Hb)
c.95 A>G (Gaohe)	II	45 (91.8, 0.68)	4 (8.2, 2.2)	0	1 (5.6, 0.60)	17 (94.4, 6.35)	0
c.392 G>T (Quingyan)	III	9 (45, 1.17)	11 (55, 2.41)	0	1 (7.7, 1.60)	12 (92.3, 6.19)	0
c.493 A>G (Taipei)	II	1 (100, 0.64)	0	0	0	0	0
c.517 T>C (Nankang)	II	1 (100, 1.39)	0	0	0	0	0
c.592 C>T (Shunde)	II	0	0	0	0	3(100, 6.23)	0
c.871 G>A (Viangchan)	II	20 (95.2, 0.48)	1 (4.8, 2.17)	0	1 (8.3, 0.51)	10 (83.4, 5.91)	1 (8.3, 9.72)
c.1004 C>A (Fushan)	II	1 (100, 1.58)	0	0	0	0	0
c.1024 C>T (Chinese-5)	III	1 (7.1, 1.52)	13 (92.9, 2.66)	0	1 (7.7, 1.14)	12 (92.3, 6.20)	0
c.1311 C>T	–	4(80.0, 1.11)	1 (20.0, 8.30)	0	1(16.7, 0.24)	5 (83.3, 5.50)	
c.1360 C>T (Union)	II	3 (100, 0.68)	0	0	0	4 (100, 4.93)	0
c.1376 G>T (Canton)	II	93 (97.9, 0.44)	2 (2.1, 1.88)	0	2 (3.8, 0.74)	49 (94.2, 6.25)	1 (1.9, 9.98)
c.1388 G>A (Kaiping)	II	122 (96.8, 0.60)	4 (3.2, 2.42)	0	1 (2.4, 0.01)	38 (90.5, 6.50)	3 (7.1, 9.83)
All compound mutations	**–**	0	0	0	24 (80.0, 0.71)	6 (20.0, 2.73)	0
All homozygous mutations	**–**	0	0	0	18 (81.8, 0.74)	4 (18.2, 3.61)	0

Median value (15.96 IU/g Hb) obtained from normal male subjects in our study considered as normal G6PD activity. The residual enzyme activity ≤ 1.6 IU/g Hb (10% of normal) was grouped into class II (severe deficiency); between 1.6–9.6 IU/g Hb (10–60% of normal) class III (moderate deficiency), and between 9.6–24.0 IU/g Hb into class IV (normal). Class I, which includes severely deficient variants associated with chronic nonspherocytic hemolytic anemia (CNSHA), was not considered in the study. Class V was not presented in the table because all residual enzyme activities in subjects with mutation detected were no more than 150% of normal.

## Discussion

In this population-based prospective study, the prevalence of G6PD deficiency and the distribution of various G6PD gene mutations, as well as the correlation between genotypes and enzyme activity phenotypes in the Chinese Han population, were well characterized through healthy adults originated from 21 provinces. The results showed that the top six frequency of G6PD gene variants in the Han Chinese were c.1376C>T, c.1388G>A, c.95A>G, c.392G>T, c.871G>A, and c.1024C>T. Most of the genetic variants identified in the Chinese population grouped into class II or III based on the G6PD activities measured in the study as well as the classification standard recommended by the WHO. The distribution of G6PD activities depended on the types of mutation patterns and genders. Compound mutations could lead to a severe deficiency of G6PD enzyme activity. These findings would provide a more accurate and comprehensive profile of G6PD deficiency in China.

In our study, the prevalence of G6PD deficiency in the Han Chinese was evaluated based on large healthy adults. The data demonstrated that the prevalence of G6PD deficiency among different regions in China was highly variable, and that in northern China was comparatively lower than that in southern China. The overall prevalence of 2.10% in the present study was comparable to that in a recent nationwide investigation, in which the overall prevalence of 2.3% was obtained from 194 screening laboratories all over the country^16^. To a great extent, these data might reflect the whole status of G6PD deficiency in China since these large enrolled populations came from diverse provinces and municipalities across the country. It should be noted, of course, that the prevalence of G6PD deficiency is imbalanced among various regions in China, and thereby that the population proportion from different provinces would have an impact on it^5,7^. Additionally, the incidence defined in every study also depended importantly on methods and reference limit used for G6PD measurement and evaluation.

Despite the great heterogeneity of G6PD gene mutations worldwide, a limited number of specific alleles were predominant in each geographical area^17^. In China, more than 35 kinds of G6PD gene mutations have been identified, and c.1388 G>A and c.1376 G>T were dominant in previous studies^5,6^. In the present study, a total of 11 pathogenic mutations were identified, of which c.1388G>A and c.1376G>T were observed with the comparable frequency and accounted for approximately 64% of all G6PD deficient alleles. The high frequency of c.1388G>A and c.1376G>T in our study was similar to previous studies in the Han Chinese population^12–15^. The c.95A>G was also an important variant in China, and its incidence varied from 8.8–14.2% in different studies^5,12,15^. In our study, this variant was identified in about 13.8% of deficient subjects, consistent with that in Lin’s and Liu’s studies^5,12^. Another three mutations, namely c.392G>T, c.871G>A, and c.1024C>T, were responsible for roughly 17% of G6PD deficiency, with a minimum frequency of 5% for each mutation. In sum, the top six most frequent pathogenic mutations accounted for more than 90% of all subjects with G6PD deficiency in our study. The overall distribution of these variants was comparable to that in a nationwide study conducted in 18 provinces and municipalities^5^. The comparison of G6PD mutation distribution found that the frequency of each variant varied between different studies^5,12,14,15^. This variation could be mainly attributed to the diverse demographic composition and molecular methods used. PCR-RBD assay used in the study covered 14 common mutations in the Chinese population and could identify more than 95% of G6PD deficiency. The results from our and other studies indicated that despite predominant variants, there might be some heterogeneity of G6PD genetic variants in the Han Chinese population.

The mutation of c.871G>A was associated with two variants: Jammu and Viangchan, which differed for the 1311 polymorphism^18^. The linkage disequilibrium between mutation 871A and polymorphism 1311 T was found in all 39 individuals carrying c.871G>A mutation in our study, suggesting that this variant was Viangchan rather than Jammu. This linkage disequilibrium on G6PD Viangchan (c.871G>A) in this study was in agreement with that in previous reports in China^9,12,15,19^ and other Asian population^20,21^.

Nucleotide position 1311(C>T) was regarded as a silent polymorphism, and could not result in the substitution at the amino acid level. The c.1311C>T polymorphism was widely detected among the G6PD deficient and normal population with various frequencies^15,22,23^. The prevalence of c.1311C>T in this study was similar to that in most of the previous studies^15,23^. We noticed that 11 G6PD deficient subjects, including six heterozygous females and five hemizygous males, were identified with only c.1311C>T polymorphism. It seemed that this polymorphism was in a position to cause the G6PD deficiency under specific conditions since the inadequacy of the testing process could not explain this finding. The significant reduction in enzyme activity for c.1311C>T polymorphism with some intronic mutations has been reported in some previous studies^6,24,25^, but intronic mutation detecting was not included in the study. The mechanism and significance of c.1311C>T polymorphism in the enzyme activity needed further to be clarified.

Although only 11 missense mutations and one polymorphism mutation were identified in our study, there existed 31 genotypes (Table 2). Most subjects enrolled in this study showed a single-site mutation. It was noteworthy that five kinds of homozygous and 11 compound heterozygous genotypes were identified in 10.2% and 14.0% of female subjects with G6PD deficiency in our study, respectively. The incidence was comparable to that in Peng’s study, but much higher than that in other studies^12,14,19^. As expected, the mutations involved in homozygous and compound heterozygous mutation patterns were dominantly associated with the G6PD-deficient alleles of c.1388 G>A or c.1376 G>T, which occurred at the highest frequencies in the Chinese population^5,7,12,14^.

It was of great importance to find out the relationship between a specific genotype and phenotype for accurately classifying subjects with some mutation patterns into deficient, moderate or normal groups, and for the management of G6PD deficiency^26,27^. To our knowledge, this is the first study to evaluate the genotype and G6PD activity phenotype in the Chinese population based on large samples. We discussed the classification of the variants identified in the study through the G6PD activities in hemizygous mutants since the inheritance of G6PD deficiency shows a typical X-linked pattern. The classification results of G6PD activities showed that all variants, except Quingyan (c.392G>T), identified in our study could be distinctly grouped into the expected class, in concordance with what has been reported before for these variants^17^. Most enzyme activities of c.95A>G, c.871G>A, c.1388 G>A, and c.1376 G>T fell into the severely deficient enzyme activity region (< 1.6 IU/g Hb), being class II. Surprisingly, the variant of Quingyan (c.392G>T) spanned both class II and III, clearly different from previous reports^17^. Our data from large samples might more accurately reveal the status of this variant affecting the G6PD activity. Among those variants within the same classification, the significant differences in G6PD activities were also observed, and the variant of 1376 G>T possessed the lowest enzyme activity in our study. Similar results were found in the previous study conducted in a small group of G6PD deficient patients of Chinese descent^28^. The study of the G6PD crystal structure revealed that the position of mutations was associated with the severity of G6PD deficiency^29^. Our findings provided more supports to the concepts that diverse variants might account for the heterogeneous activity phenotype of G6PD deficiency. It was noticed that a small part of the activities in some variants fell outside the expected range of G6PD activity. This bias seemed inevitable in clinically experimental operations, as many factors might have an impact on the results of G6PD activity measurements. We speculated that this might be attributed to individual heterogeneity since this bias presented in almost every variant. Additionally, the error in the testing procedure and unit conversion might also contribute to this. Despite the classification difference and individual heterogeneity, in our study, all enzyme activity values in hemizygous males for every variant were within a narrow range under 30% of the normal activity, far from the cutoff value. The phenomenon that the G6PD activity range in hemizygous males with A- variant stretched into the borderline of cutoff value was not observed in the variants identified in our study^27^. This disparity might be attributed to the diverse types of mutations in different ethnic population. Compared with hemizygous males, much wider distribution of enzyme activities, from severely deficient to near the cutoff value, was revealed in heterozygous females with the same variant, although the G6PD activity in single heterozygous females mainly demonstrated a moderate deficiency within 30–60% of the normal activity, falling into class III. Furthermore, mutations were also identified in G6PD normal females. Thus, G6PD activities in single heterozygous females spanned across all classes of the deficient regions and into the region of normal activity. This distribution feature of G6PD activity in females was mainly attributed to the genetic mosaics as a result of X-chromosome inactivation^2,3^. Although red blood cells would be expected to have normal G6PD activity in 50% of cases resulting in an intermediate activity, the inactivation is often skewed^30^, producing the activity from hemizygous-like deficient to normal. The degree to which a heterozygous female had G6PD deficiency depended on the proportion of red cells expressing the normal or deficient enzyme. Our data showed that few females tended severe G6PD deficiency.

It was well known that hemizygous males and homozygous females typically had much lower G6PD activity than heterozygous females^2,3^. This was further confirmed in our study, in which 22 homozygous females were identified with relatively low enzyme activity. The mean value and distribution range of G6PD activities in homozygous females in our study was comparable to that in hemizygous males, but was significantly lower than that in single heterozygous females. To date, few studies examined the phenotype-genotype correlations in the mutants with more than one missense mutation, although compound heterozygous mutants were not rare in the Chinese population^12,14,15,19^. In our study, 30 compound heterozygous mutants, possessing 12% of all deficient females, provided significant insight into the relationship between enzyme activity and the compound mutation process in the G6PD gene. Our data showed that, compared with single heterozygous mutants, G6PD activity in compound heterozygous mutants reduced dramatically to the comparable level of that in homozygous mutants; and this decrease was found in any combination of two mutations. This remarkable decline of G6PD activity in females with double mutations was also reported in a study by Nantakomol^31^. These findings allowed us to support the hypothesis that G6PD genotypes with two missense mutations confer a remarkable reduction in G6PD activity than mutants with only a single missense mutation in females. Studies by Boonyuen et al. demonstrated that the combination of two mutations had a synergistic effect on decreasing the catalytic activity and/or protein stability, depending on the kinds of mutations for the combination^32,33^. The clinical significance of the compound mutations needs to be further elucidated. The presence of these compound mutants added further complexity to the phenotypic test of G6PD deficiency as the severity of clinical manifestations might depend more on the genotype, more than only enzyme activity^34,35^.

Hence our data analysis showed that the distribution feature of G6PD activities depended on the type of mutation patterns and genders. Based on the distribution of G6PD activity in the Chinese population, a significant gap between the G6PD activity in hemizygous males and normal males was observed. A similar gap was also presented in homozygous or compound heterozygous females and normal females, but not in single mutation heterozygous females and normal females. The overall distribution of G6PD activities in females carrying missense mutations was a continuum from G6PD severely deficient to normal. These findings were consistent with that in Denmark’s study in the adult population^36^, and also with that in neonates in previous studies^37,38^.

In summary, the nationwide population-based G6PD pathogenic mutation distribution profile in the Han Chinese people was well characterized in this study, and for the first time, the relationship between the classification and distribution of enzyme activity and mutation patterns as well as genders was clearly revealed through the analysis of G6PD activities from a large population. The study should be very informative for the management of G6PD deficiency, and would provide some data for future studies on the relationship between genotype and clinical phenotype as the studies showed that the severity of the haemolytic crisis in the population with specific mutations was independent of the residual enzymatic activity^34,35^.

## Materials and methods

### Study design and subjects

Shenzhen is a typical immigrant city, and most of its population come from the immigration of the other provinces of China outside Guangdong province. Taking advantage of this demographic source characteristic, we performed a three years’ study from October 2016 to October 2019 and attempted to identify the overall prevalence and molecular profile of G6PD deficiency in China. The couples who visited hospitals for premarital medical examination and genetic counseling were enrolled in the study. Basic demographic information was recorded. All subjects with G6PD deficiency by a phenotypic method in the second study year were selected to screen for G6PD gene variants by PCR and reverse dot blot (PCR-RDB) assay. If the common mutations were not identified, gene sequencing analysis was performed. A similar number of males and females with normal G6PD activity were randomly selected for mutation detection by the PCR-RDB assay. The study was approved by the Medical Ethics Committee of the Fourth People's Hospital of Shenzhen (now is renamed the Eighth Affiliated Hospital, Sun Yat-sen University) and all methods were carried out in accordance with relevant guidelines and regulations. Written informed consent was obtained from all subjects.

### Quantitative assay of G6PD activity

G6PD enzyme activity was measured by a quantitative spectrophotometric assay based on the evaluation of absorbance at 340 nm given by NADPH formation. Whole blood samples were collected into tubes containing EDTA-K2. The haemogram analysis was done using an automated hematology analyzer (Beckman Coulter, USA). After purified erythrocytes were lysed, the G6PD activity was determined by commercially available kits (Guangzhou Kofa Biotechnology, Guangdong, China) on Hitachi Model 7600 Series Automatic Analyzer (Hitachi High-Technologies Corporation, Ibaraki, Japan) and AU5800 automatic biochemistry analyzer (Beckman Coulter, USA). The operations were followed by the manufacturer’s instructions. The cutoff value for G6PD deficiency was set at 10.0 IU/g Hb according to the manufacturer’s recommendation. All samples were assayed within 24 h after sample collection to prevent the reduction of G6PD activity. Samples in the presence of obvious hemolysis, abnormal erythrocyte count were excluded^39^. G6PD activity was finally expressed as the ratio of G6PD units per gram of Hb.

### Molecular diagnosis of G6PD deficiency

Mutations were identified through PCR-RDB assay combined with flow-through hybridization technology platform and kit (Hybribio Chaozhou Co., Ltd.), which has been described in detail previously^23^. In brief, DNA was purified from leucocytes using DNA extraction system and kit (Hybribio Chaozhou Co., Ltd, Guangdong, China), followed by 40 cycles of 95 °C for 30 s, 55 °C for 30 s, 72 °C for 45 s, and a final extension at 72 °C for 15 min. The amplification was performed on an MJ Mini Personal Thermal Cycler (Bio-RAD, USA). Two separate reaction systems were respectively used in two tubes, the primers of one system consisted of Exon2-F, Exon2-R, Exon5-F, Exon5-R, Exon 6-F, and Exon 6-R, while the primers in another system included Exon9-F, Exon9-R, Exon11-12-F and Exon11-12-R. The amplicons of the two reaction systems (two tubes) were subsequently denatured at 95 °C for 8 min and immediately submitted to hybridization.

The hybridization reactions were performed on the Hybrid instrument HB-2012A (Hybribio Chaozhou Co., Ltd.). Briefly, all probes were immobilized on a nylon membrane with a fixed dot matrix. After membrane fibers and hybridization solution incubated at 45 °C for 5 min, the mixture of PCR products in 0.8 ml of preheated hybridization solution was added into the membrane fibers, then flow-through hybridized at 45 °C for 30 min. After a stringent wash at 45 °C, the hybrids were detected by the addition of the streptavidin–horseradish peroxidase conjugate, which bound to the biotinylated PCR products and a substrate NBT/BCIP to generate a blue-purple precipitate at the probe dot. The positive and negative controls provided with the kit were included in each hybridization to assess the performance of the test. The positive results were interpreted by direct visualization of blue-purple dots. Using this *G6PD* gene detection kit, fourteen common mutations in the Chinese population accounting for more than 95% of G6PD deficiency could be identified.

### G6PD gene sequencing

The samples with decreased enzyme activity, if no mutation was detected by the aforementioned assay, were further analyzed by DNA sequencing for the coding region (exon 2 to exon 13). In brief, after purified using the QiagenDyeEx 2.0 Spin Kit (QIAGEN China Co., Ltd., Shanghai, China), PCR products were submitted to the INVITROGEN biological technology company (Shanghai, China) for sequence analysis with BigDye Terminator kits and ABI 3500DX Genetic Analyser (Applied Biosystems, USA). The detailed information of primer pairs has been described in the previous study^40^.

### Statistical analysis

Before statistical analysis was performed, the distribution status of data was evaluated to choose an optimal statistical method. Variables such as age, detection rate were described by descriptive statistics. A chi-square test was used for comparison of frequencies of G6PD deficiency between males and females. The Mann–Whitney U-test was used for non-parametric comparisons. A *p*-value < 0.05 was considered statistically significant. Statistical analysis was conducted with the SPSS 23.0 Statistical Software package.
